# Characterization of a Ginsenoside-Transforming β-glucosidase from *Paenibacillus mucilaginosus* and Its Application for Enhanced Production of Minor Ginsenoside F_2_

**DOI:** 10.1371/journal.pone.0085727

**Published:** 2014-01-27

**Authors:** Chang-Hao Cui, Jin-Kwang Kim, Sun-Chang Kim, Wan-Taek Im

**Affiliations:** 1 Department of Biological Sciences, Korea Advanced Institute of Science and Technology, Yuseong-gu, Daejeon, Republic of Korea; 2 KAIST Institute for Biocentury, Korea Advanced Institute of Science and Technology, Yuseong-gu, Daejeon, Republic of Korea; 3 Intelligent Synthetic Biology Center, Yuseong-gu, Daejeon, Republic of Korea; University of Nottingham, United Kingdom

## Abstract

A novel β-glucosidase (BglPm) was identified from *Paenibacillus mucilaginosus* KCTC 3870^T^ which has ginsenoside converting activity. The gene, termed *bglPm*, consists of 1,260 bp and belongs to glycoside hydrolase family 1 (GH1). After being overexpressed and purified from *Escherichia coli*, the enzymatic properties of BglPm were investigated. The enzyme exhibited an optimal activity at 45°C and pH 7.5 and showed high bioconversion ability for major ginsenoside Rb_1_ and Rd into ginsenoside F_2_. Thus, it was used for mass production of relatively high pure F_2_ from relatively abundant protopanaxadiol type ginsenosides mixture (PPDGM) with combined usage of ginsenoside Rc-hydrolyzing enzyme. Scale-up of production using 250 g of the PPDGM resulted in 152 g of F_2_ with 80.1% chromatography purity and 95.7% recovery. These results suggest that this enzyme would be useful in the preparation of pharmacologically active ginsenoside F_2_ in the functional food and pharmaceutical industries.

## Introduction

The root of *Panax ginseng* C.A. Meyer is commonly used in Northeast Asian countries as a traditional medicine for more than 2000 years and has become better known in the West during the past few decades [1], [2], [3]. Therapeutic effects of ginseng such as anti-neoplastic, anti-stress, and anti-oxidant activities have been attributed mainly to the primary active ingredients-ginsenosides [4], [5], [6], [7]. More than 180 different ginsenosides have been identified, and they can be categorized as protopanaxadiol (PPD), protopanaxatriol (PPT), and oleanane saponins based on the structure of the aglycon [5], [6], [8]. Ginsenosides Rb_1_, Rb_2_, Rc, Rd, Re, and Rg_1_ are major ginsenosides that make up more than 80% of total ginseng ginsenosides [4], [5], [9], and minor ginsenosides (Rg_3_, Rh_2_, F_2_, C-K, Rg_2_, Rh_1_, and F_1_) that are deglycosylated from the major ginsenosides exist in smaller amounts or are absent in ginseng. The deglycosylated minor ginsenosides have some chemical reactivity that the major ginsenosides do not. Furthermore, emerging evidence has demonstrated that the minor ginsenosides have more important pharmaceutical effects, such as anti-cancer, anti-diabetic, anti-oxidative, and anti-aging effects, than the glycosylated major ginsenosides [10], [11], [12], [13].

As a minor ginsenoside, F_2_ accounts for less than 0.01% in raw ginseng and red ginseng (a heat-treated ginseng with more minor ginsenosides) [14], and thus isolation of F_2_ from natural products is difficult. F_2_ has been produced via bioconversion of PPD type ginsenosides [i.e. Rb_1_, gypenoside XVII (Gyp XVII), Rd etc.] using fungal β-glucosidase or recombinant β-glucosidase derived from bacteria [15], [16]. Owing to the difficulty of usage of research material, a few pharmaceutical activities have thus far been surveyed using F_2_, which was also gained using biotransformation. F_2_ exerted effects against malignant brain tumor and breast cancer stem cells [17], [18]. Thus, it is imperative to develop mass production of F_2_ for its application as a functional material for cosmetics, functional health supplements, and drugs. Although some researchers have identified ginsenoside bioconversion enzymes which can produce F_2_ from major ginsenosides [19], they only conducted simple enzyme characterizations without further scale-up or process engineering.

Attempts to produce gram-scale ginsenosides have been made using microbial method. The major ginsenoside Rd has been produced on a gram-scale from the pure ginsenoside Rb_1_ using *Paecilomyces bainier* 229-7 [20]. Thus, it is timely to design and develop a means of mass production of minor ginsenosides to meet industrial demand and fulfill their original purpose of application as a recombinant enzyme. Recently, minor ginsenoside Rg_3_(*S*) was produced successfully as 100 g unit with relatively high purity using two recombinant glycoside hydrolases in series by our group [21].

In the present study, a ginsenoside-transforming β-glucosidase was cloned from *Paenibacillus mucilaginosus* KCTC 3870^T^. The recombinant protein, BglPm, was purified and the enzymatic properties were investigated. This enzyme showed strong ginsenoside-transformation ability, especially major ginsenoside Rb_1_ and Rd into minor ginsenoside F_2_. Furthermore, enhanced production of F_2_ from relatively abundant protopanaxadiol type ginsenosides mixture (PPDGM) from ginseng extraction was performed using recombinant BglPm and another α-L-arabinofuranosidase (Abf22-3) with ginsenoside-Rc transformation activity from *Leuconostoc* sp. 22-3 which has been cloned by our group [22]. BglPm displayed excellent F_2_-production activities and can be used for mass production of relatively pure compound from abundant PPDGM and may prompt the pharmacological studies and applications of rare ginsenoside F_2_.

## Methods

### 2.1. Materials

The PPD type ginsenosides mixture (PPDGM) from the root of *Panax quinquefolius* [comprised of Rb_1_: 53.8%, Rc: 15.8%, Rb_2_: 2.8%, Rb_3_: 4.8%, Rd: 16.7%, Rg_3_(*S*): 1.7% and other ginsenosides: 4.4% based on mole ratio] from Hongjiou Biotech Co. Ltd. (China) was used as the substrate in the current investigation. Ginsenosides standards which are over 98% purity such as Rb_1_, Rc, Rb_2_, Rd, Rg_3_(*S*), Rh_2_(*S*), F_2_, compound K (C-K), PPD, Rg_1_, Re, Rg_2_(*S*), Rh_1_(*S*) and PPT were purchased from Nanjing Zelang Medical Technology Co., Ltd. (China). Methanol and acetonitrile with HPLC grade were obtained from Merck (Darmstadt, Germany). Gypenoside XVII (Gyp XVII), compound O (C-O), compound Mc_1_ (C-Mc_1_), and compound Mx1 (C-Mx_1_) were prepared as described by An et al. (2010) and Wang et al. (2011). The other chemicals used in this study were a minimum of analytical reagent grade, and the sources are noted individually in the Methods section. Recombinant α-L-arabinofuranoside (Abf22-3) was prepared as described [22]. The genomic DNA from *Paenibacillus mucilaginosus* KCTC 3870^T^, *Escherichia coli* BL21 (DE3), and pGEX 4T-1 plasmid (GE Healthcare, USA) were used as β-glucosidase gene, host, and expression vector sources, respectively. *P. mucilaginosus* KCTC 3870^T^ was grown in aerobic conditions at 37°C on nutrient agar (NA, BD, USA). The recombinant *E. coli* for protein expression was cultivated in a Luria-Bertani (LB) medium supplemented with ampicillin (100 mg/l).

### 2.2. Analysis of BglPm sequence

Database homology search was performed with BLAST program provided by NCBI. Furthermore, the multiple amino acid sequence alignment and the conserved patterns of discrete amino acid sequences of BglPm and known the most homologous β-glucosidases were performed by using ClustalW program (http://embnet.vital-it.ch/software/ClustalW.html).

### 2.3. Molecular cloning, expression, and purification of recombinant BglPm

The genomic DNA from *P. mucilaginosus* KCTC 3870^T^ was extracted using a genomic DNA extraction kit (Solgent, Korea). The gene encoding β-glucosidase was amplified from the genomic DNA as a template via a polymerase chain reaction (PCR) using *Pfu* DNA polymerase (Solgent, Korea). The sequence of the oligonucleotide primers used for the gene cloning was based on the DNA sequence of β-glucosidase (GenBank accession number: **AEI42200**). Forward (5′- **G GTT CCG CGT GGA TCC GAA TAT ATT TTT CCA CAG CAA TTT** -3′) and reverse (5′- **G ATG CGG CCG CTC GAG TTA CAG CAC TTT CGT GGA TGC GAT** -3′) primers were designed as primers to introduce the BamHI and XhoI restriction sites (underlined), respectively, and were synthesized by Macrogen Co. Ltd. (Korea). The amplified DNA fragment obtained from the PCR was purified and inserted into the pGEX 4T-1 GST fusion vector digested with BamHI and XhoI using an EzCloning Kit (Enzynomics Co. Ltd., Korea). The resulting recombinant pGEX-*bglPm* was transformed into *E. coli* BL21(DE3). The *E. coli* BL21(DE3) harboring the recombinant plasmid was grown in an LB-ampicillin medium at 37°C until the culture reached an OD_600_ of 0.6, at which point the protein expression was induced through the addition of 0.1 mM isopropyl-β-D-thiogalactopyranoside (IPTG). The bacterial cells were incubated for a further 18 h at 30°C and were then harvested via centrifugation at 13,000 rpm for 15 min at 4°C. The cells were washed twice with a solution consisting of 50 mM sodium phosphate, 5 mM EDTA, and 1% Triton X-100 (pH 7.0); then, they were resuspended in 50 mM sodium phosphate (pH 7.0). The cells were disrupted via ultrasonication (Vibra-cell, Sonics & Materials, CT, USA). The intact cells and debris were removed via centrifugation at 13,000 rpm for 15 min at 4°C in order to obtain the crude cell extract. The GST tag was purified using the GST·bind agarose resin (Elpisbiotech Co. Ltd, Korea). The homogeneity of the protein was assessed using 10% SDS-PAGE and an EZ-Gel staining solution (Daeillab Co. Ltd., Korea).

### 2.4. Effect of pH, temperature, metal ions and chemical reagent on enzyme activity

The specific activity of purified BglPm was determined using p-nitrophenyl-β-D-glucopyranoside (pNPGlc) as a surrogate substrate in 50 mM sodium phosphate buffer, pH 7.5 at 37°C. Reactions were stopped after 10 minutes (min) by the addition of Na_2_CO_3_ at a final concentration of 0.5 M, and the release of p-nitrophenol was measured immediately using a microplate reader at 405 nm (Bio-Rad model 680; Bio-Rad, Hercules, CA). One unit of activity was defined as the amount of protein required to generate 1 µmol of p-nitrophenol per min. Specific activity was expressed as units per milligram of protein. Protein concentrations were determined using the bicinchoninic acid (BCA) protein assay (Pierce, Rockford, IL), with bovine serum albumin (Sigma) as the standard. All assays were performed in triplicate.

The effect of pH on enzymatic activity was determined using 2.0 mM pNPGlc as a substrate in the following buffers (each at 50 mM): KCl-HCl (pH 2.0), glycine-HCl (pH 3.0), sodium acetate (pH 4.0 and 5.0), sodium phosphate (pH 6.0, 7.0 and 7.5), Tris-HCl (pH 8.0, and 9.0) and glycine-sodium hydroxide (pH 10). The pH stability of recombinant BglPm was determined by measuring enzymatic activity after incubation in each buffer (containing 2.0 mM pNPGlc in 50 mM potassium buffer as a substrate) for 12 h at 4°C. The results are expressed as a percentage of the activity obtained at the optimum pH. The effect of temperature on enzymatic activity was tested by incubating the enzyme at various temperatures ranging from 4 to 65°C at optimum pH for 10 min in 50 mM potassium phosphate buffer containing 2.0 mM pNPGlc. The thermostability of the enzyme was examined by incubating the enzyme in 50 mM potassium phosphate buffer for 30 min at different temperatures. After cooling the sample on ice for 10 min, activity was determined using pNPGlc as the substrate.

The effects of metals and other chemicals on BglPm activity were also determined. BglPm activity was tested in the presence of 1 or 10 mM (final concentration) of HgCl_2_, MnCl_2_, CaCl_2_, CoCl_2_, MgCl_2_, EDTA, NaCl, KCl, CuCl_2_, SDS, dithiothreitol (DTT), or β-mercaptoethanol for 30 min at 37°C. The remaining activity was determined using pNPGlc as a substrate, and activities are expressed as a percentage of the activity obtained in the absence of the compound.

Substrate preference was examined using 2.0 mM chromogenic o-nitrophenyl (ONP) and p-nitrophenyl (PNP) as substrates at 37°C for 10 min, with one activity unit being defined as the release of 1 µmol o-nitrophenol or p-nitrophenol per min. The following substrates were tested: PNP-β-D-glucopyranoside, PNP-β-D-galactopyranoside, PNP-β-D-fucopyranoside, PNP-N-acetyl-β-D-glucosaminide, PNP-β-L-arabinopyranoside, PNP-β-D-mannopyranoside, PNP-β-D-xylopyranoside, PNP-α-D-glucopyranoside, PNP-α-L-arabinofuranoside, PNP-α-L-arabinopyranoside, PNP-α-L-rhamnopyranoside, PNP-α-D-mannopyranoside, PNP-α-D-xylopyranoside, ONP-β-D-glucopyranoside, ONP-β-D-galactopyranoside, ONP-β-D-fucopyranoside and ONP-α-D-galactopyranoside (all from Sigma).

### 2.5. Determination of kinetic parameters

Kinetic studies were performed with freshly purified enzymes using pNPGlc at 0.1–10.0 mM, Rb_1_, Gyp XVII and Rd at concentrations from 0.2 mM to 5.0 mM. One unit of activity was defined as the amount of protein required to generate 1 µmol of p-nitrophenol or to convert 1 µmol of Rb_1_ or Gyp XVII or Rd per minute. All assays were performed in triplicate. The parameters, *K_m_* and *V_max_*, were determined using the enzyme kinetics program described by Cleland [23].

### 2.6. Biotransformation activity of PPD ginsenosides using BglPm

The initial biotransformation experiments using the major ginsenosides Rb_1_ and Rd as substrates revealed that the GST-fused enzyme does not affect the activities of BglPm. Therefore, the fusion protein (GST-BglPm) was used to determine the specificity and selectivity of the enzymes for the hydrolysis of the glucose moieties attached at the C3 and C20 sites in the seven PPD ginsenosides. The enzyme solutions at a concentration of 0.1 mg/ml in 50 mM of sodium phosphate buffer (pH 7.5) were reacted with an equal volume of Rb_1_, Rb_2_, Rb_3_, Rc, Rd, Gyp XVII and Rg_3_(*S*) solution at a concentration of 0.1% (wt/vol) in 50 mM of sodium phosphate buffer (pH 7.5) at 37°C. The samples were taken at regular intervals and analyzed via TLC or HPLC after pretreatment (see analytic methods).

### 2.7. Optimization of concentration of the substrate

In order to determine the optimal condition for the biotransformation of PPDGM to F_2_, the substrate concentration of PPDGM in the reaction was optimized. The final crude BglPm concentration was fixed to 10 mg/ml (32 U/ml of pNPGlc activity) and reacted with an equal volume of 20, 50, 100 and 150 mg/ml of PPDGM in order to have 10, 25, 50 and 75 mg/ml as the final substrate concentrations. These 4 types of optimization reactions were performed in a 50 ml conical tube with a 10 ml working volume at 150 rpm for 12 h at 37°C. The samples were taken at regular intervals and analyzed via HPLC.

### 2.8. Scaled-up biotransformation of PPDGM

#### 2.8.1. Preparation of two recombinant enzymes using high cell density culture

For the production of recombinant Abf22-3, a Luria-Bertani (LB) medium supplemented by ampicillin (100 µg/ml) was used for the cultivation of *E. coli* harboring pMBP-*abf22-3* in a 10 L stirred-tank reactor (Biotron GX, Hanil science Co., Korea) with a 5 L working volume at 500 rpm. The pH value of the medium was adjusted to be 7.0 using 100 mM sodium phosphate buffer. The culture was incubated at 37°C until the culture reached an OD of 3.0 at 600 nm. The protein expression was induced through the addition of isopropyl-β-D-thiogalactopyranoside (IPTG) with a final concentration of 0.1 mM with feeding 2% (w/v) glucose. The bacterial cells were incubated for a further 24 h at 18°C and were then harvested via centrifugation at 5,000 rpm for 20 min (Component R, Hanil science Co Ltd., Korea) at 4°C.

For the production of the recombinant BglPm, the LB medium supplemented with ampicillin (100 µg/ml) was used to cultivate the *E. coli* harboring pGEX-*bglPm* in a 10 L stirred-tank reactor (Biotron GX, Hanil science Co., Korea) with a 5 L working volume at 500 rpm. The pH value of the medium was adjusted to 7.0 using 100 mM of sodium phosphate. The culture was incubated at 37°C until the culture reached an OD of 3.0 at 600 nm. The protein expression was induced through the addition IPTG with a final concentration of 0.1 mM. The bacterial cells were incubated for a further 18 h at 30°C and were then harvested via centrifugation at 5,000 rpm for 20 min (Component R, Hanil science Co Ltd., Korea) at 4°C.

The cells suspended in 100 mM of phosphate buffer (pH 6.0 for Araf22-3 and pH 7.5 for BglPm) were disrupted via sonication (Vibra-cell, Sonics & Materials, CT, USA), and then the intact cells and debris were removed via centrifugation at 5,000 rpm for 20 min (Component R, Hanil science Co Ltd., Korea) at 4°C in order to obtain the supernatants of the crude enzymes. The crude recombinant Abf22-3 was diluted to the desired concentration with 100 mM of sodium phosphate buffer (pH 6.0) and was used to biotransform the PPDGM. The crude recombinant BglPm was lyophilized for application in the biotransformation reactor following the reaction with the recombinant Abf22-3.

#### 2.8.2. PPDGM transformation via crude Abf22-3 and BglPm in series

The scaled-up biotransformation was performed in a 10 L stirred-tank reactor (Biotron GX, Hanil science Co., Korea) with a 5.0 L working volume at 50 rpm for 24 h. The reaction was performed under optimal conditions in pH 6.0 at 37°C. The reaction started with a composition of 50 mg/ml of substrate ginsenosides (PPDGM; total 250 g) as final concentration and 1.0 L of crude recombinant Abf22-3 (10 mg/ml) in 100 mM of phosphate buffer (pH 6.0). After 6 hours when the ginsenoside Rc was nearly completed converted to Rd, pH was adjusted to 7.5 using 0.5 M NaOH and lyophilized recombinant BglPm was added. Samples were collected at regular intervals and were analyzed by HPLC in order to determine the biotransformation of the ginsenoside F_2_ from Rb_1_, Rc, and Rd.

### 2.9. Purification of biotransformed F_2_

Following the 5 L reaction of PPDGM with Abf22-3 and BglPm, the mixture was cooled at 4°C and centrifuged at 5,000 rpm for 15 min (Component R, Hanil science Co Ltd., Korea). The biotransformed ginsenoside F_2_ in the supernatants and precipitates was processed separately in order to purify the samples. The precipitate was also dissolved in 5.0 L of 70% ethanol solution twice and filtered through a filter paper (Advantec, Japan). The ethanol extracts were combined and adjusted to be a 45% ethanol solution. The column chromatography [3,170(L)×128(D) mm; Doointech, Korea] packed with HP20 resin (Mitsubishi, Japan) was adopted in order to remove the impurities, except the ginsenosides. The supernatants and 45% ethanol solution were loaded onto the column together. The free sugar molecules and unwanted hydrophilic compounds from the HP-20 that were adsorbed in beads were washed with 6 bed volumes (BV) of water, and finally the adsorbed ginsenosides were eluted using 6 BV of 95% ethanol (extra pure grade; SK Chemicals, Korea). The ethanol eluent was evaporated *in vacuo*. The resulting powder was dissolved in 100% methanol and analyzed via HPLC.

### 2.10. Analytic methods

#### 2.10.1. Thin layer chromatography (TLC) analysis

The thin layer chromatography (TLC) was performed using 60F_254_ silica gel plates (Merck, Germany) with CHCl_3_-CH_3_OH-H_2_O (65∶35∶10, lower phase) as the solvent. The spots on the TLC plates were identified through comparisons with standard ginsenoside after visualization was made by spraying 10% (vol/vol) H_2_SO_4_, followed by heating at 110°C for 5 min.

#### 2.10.2. High performance liquid chromatography (HPLC) analysis

The HPLC analysis of the ginsenosides was performed using an HPLC system (Younglin Co. Ltd, Korea) with a quaternary pump, automatic injector, single wavelength UV detector (model 730D), and Younglin's AutoChro 3000 software for peak identification and integration. The separation was performed on a Prodigy ODS(2) C_18_ column (5 µm, 150×4.6 mm i.d.; Phenomenex, USA) with a guard column (Eclipse XDB C_18_, 5 µm, 12.5×4.6 mm i.d.). The mobile phases were A (acetonitrile) and B (water). The gradient elution started with 32% solvent A and 68% solvent B, and was changed to the following: from 0–8 min, A was increased from 32 to 65%; from 8–12 min, A was increased from 65 to 100%; from 12–15 min, A was constant at 100%; from 15–15.1 min, A was decreased from 100 to 32%; from 15.1–25 min, A was constant at 32% The flow rate was 1.0 ml/min, and the detection was performed by monitoring the absorbance at 203 nm and with an injected volume of 25 µl.

## Results and Discussion

### 3.1. Analysis of BglPm sequence

**Figure 1:**
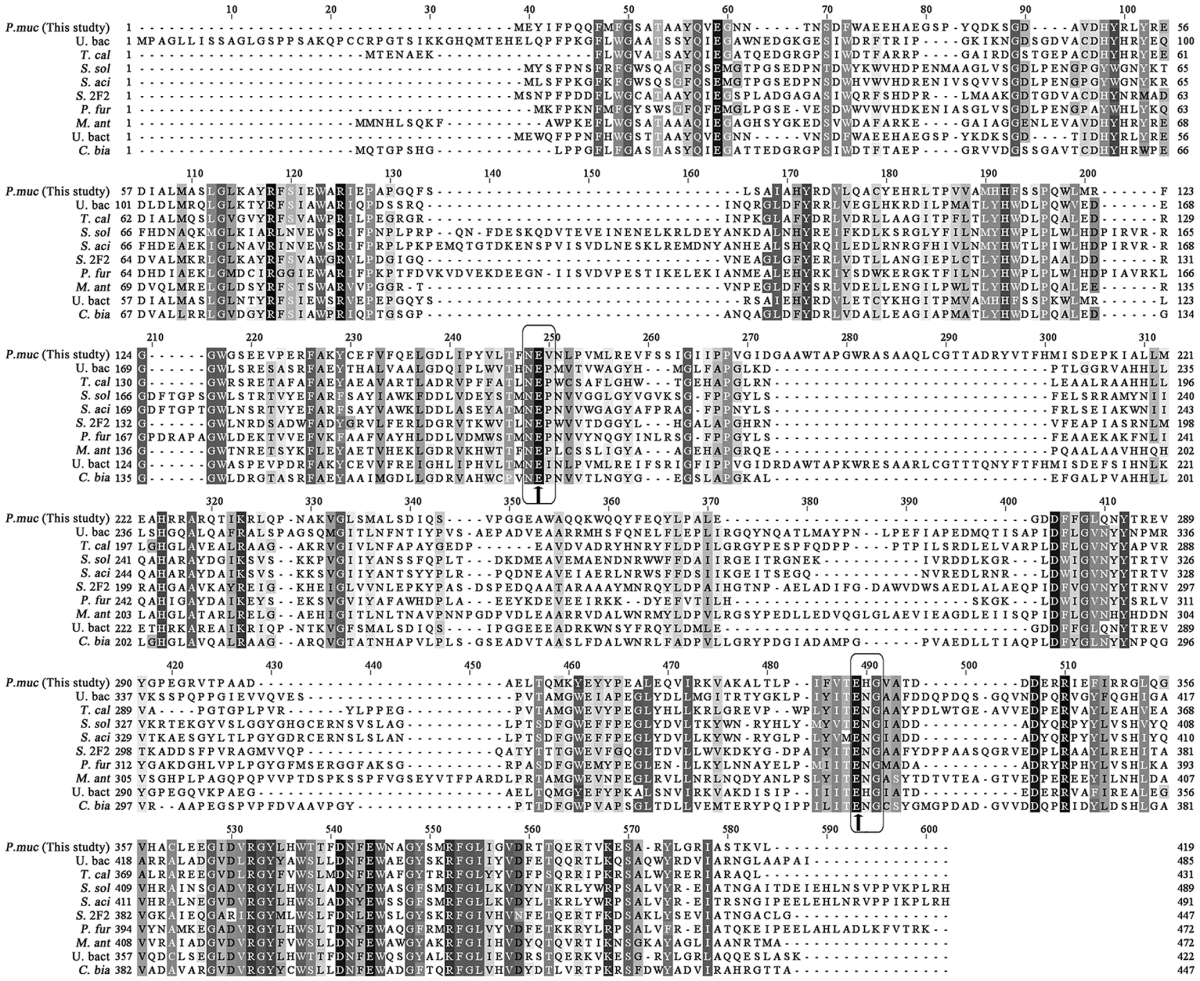
Sequence alignment of BglPm and related ginsenoside-transforming or characterized glycoside hydrolase family 1 enzymes. The conserved regions for the catalytic central in glycoside hydrolase family 1 are boxed, and the conserved catalytic amino acids are marked with an asterisk. The two putative conserved motifs were shown in box, and the predicted GH1 active site residues (general acid/base and nucleophile residue) were marked by an arrowhead. Full species names and Genbank IDs of the glycoside hydrolases family 1 are as follows, *Paenibacillus mucilaginosus* KCTC 3870^T^ β-glucosidase [*P. muc* (This study)], AEI42200; Uncultured bacteria β-glucosidase (U. bac), ABI18350; *Thermus caldophilus* β-glycosidase (*T. cal*), AAO15361; *Sulfolobus solfataricus* P2 β-glycosidase (*S. sol*), AAK43121; *Sulfolobus acidocaldarius* β-glycosidase (*S. aci*), WP_011278657; *Sphingomonas* sp. 2F2 β-glucosidase (*S.* 2F2), ADY18331; *Pyrococcus furiosus* β-glucosidase (*P. fur*), AAC25555; *Micrococcus antarcticus* β-glucosidase (*M. ant*), ACM66669; Uncultured bacterium β-glucosidase (U. bact), AFN70956; *Cellulomonas biazotea* cellobiase (*C. bia*), AEM45802.

The β-glucosidase gene (*bglPm*) consisting of 1,260 bp encoding 419 amino acids with a molecular mass of 47.72 KDa and a theoretical pI value of 5.27 (http://web.expasy.org/compute_pi/). BglPm has homology to the protein domain of glycoside hydrolase family 1 (GH1). The Carbohydrate-Active enZymes database (http://www.cazy.org) describes more than 3,000 uncharacterized and 271 characterized GH1 members that are widespread across numerous organisms. In characterized GH1 members, a protein blast (Blastp) search in the databases of NCBI indicated that the protein has the highest similarity (74%) with a β-glucosidase from metagenomic library of mangrove soil. Multiple sequence alignments of BglPm with ginsenoside-transforming and characterized glycoside hydrolases from GH1 allowed the identification of the active site (Fig. 1). Amino acid sequence comparisons revealed that BglPm shared many conserved amino acid residues with other known glycoside hydrolases from GH1, which confirmed that BglPm belonged to GH1, and they all shared the same catalytic central conserved regions (NEP and ENG domains, Fig. 1). Furthermore, Glu 158 and Glu 335, which located in both conserved regions was thought to be the active site of BglPm (Fig. 1, the arrowhead denoted the active site). Glu 158 was identified as the acid base catalyst, while Glu 335 corresponded to the catalytically active nucleophile compared with other known family 1 glycoside hydrolases.

### 3.2. Overexpression, and purification of recombinant BglPm

**Figure 2:**
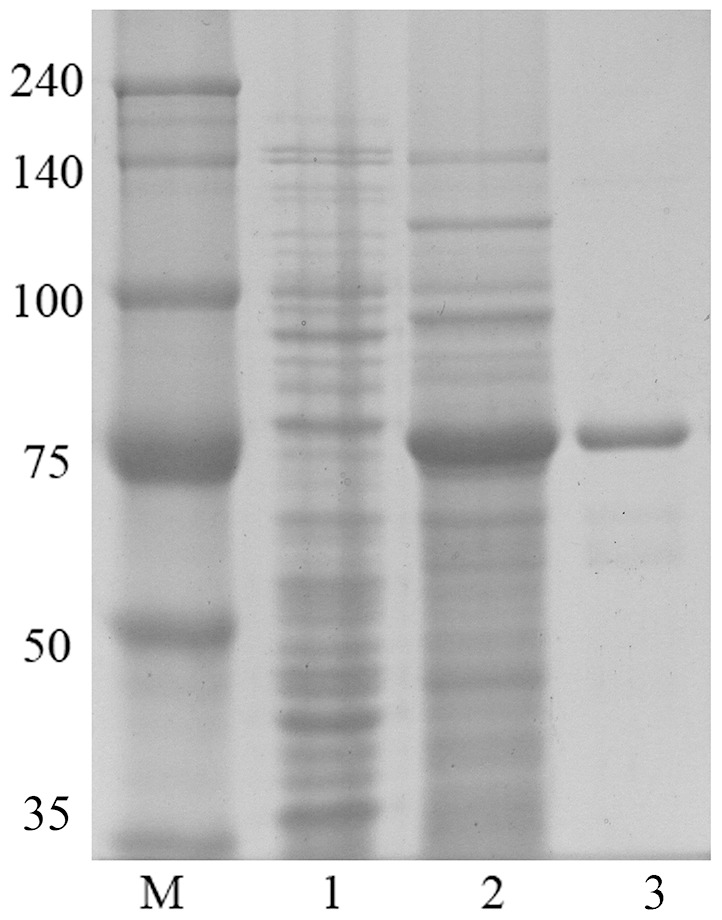
SDS-PAGE analysis of the recombinant BglPm after purification using the GST-bind agarose resin. Lanes: M, molecular weight standard; 1, insoluble fraction of the crude extract of the induced recombinant BL 21 (DE3) cells; 2, soluble fraction of the crude extract of the induced recombinant BL 21 (DE3); 3, GST-BglPm after purification with the GST-bind agarose resin.

The *bglPm* was amplified via PCR and then inserted into the pGEX 4T-1 vector. In order to maximize the yield of the fusion protein in a soluble form, different induction conditions were tested and it was found that an induction with 0.1 mM IPTG at 30°C for 18 h cultivation after induction produced the maximum level of soluble active fusion enzyme (data not shown). The recombinant enzyme was purified by GST•bind agarose resin, and then supernatant from cell lysates as well as purified protein were applied to SDS–PAGE (Fig. 2). The molecular mass of the GST-BglPm calculated via an amino acid sequence was 74.6 Kda which is similar masses with the migration in SDS-PAGE. In addition, the recombinant GST-BglPm contains 42.5±0.3% of total soluble protein in *E. coli* lysate. This high expression level in soluble form makes it more possible to industrial application.

### 3.3. Characterization of recombinant BglPm

**Figure 3:**
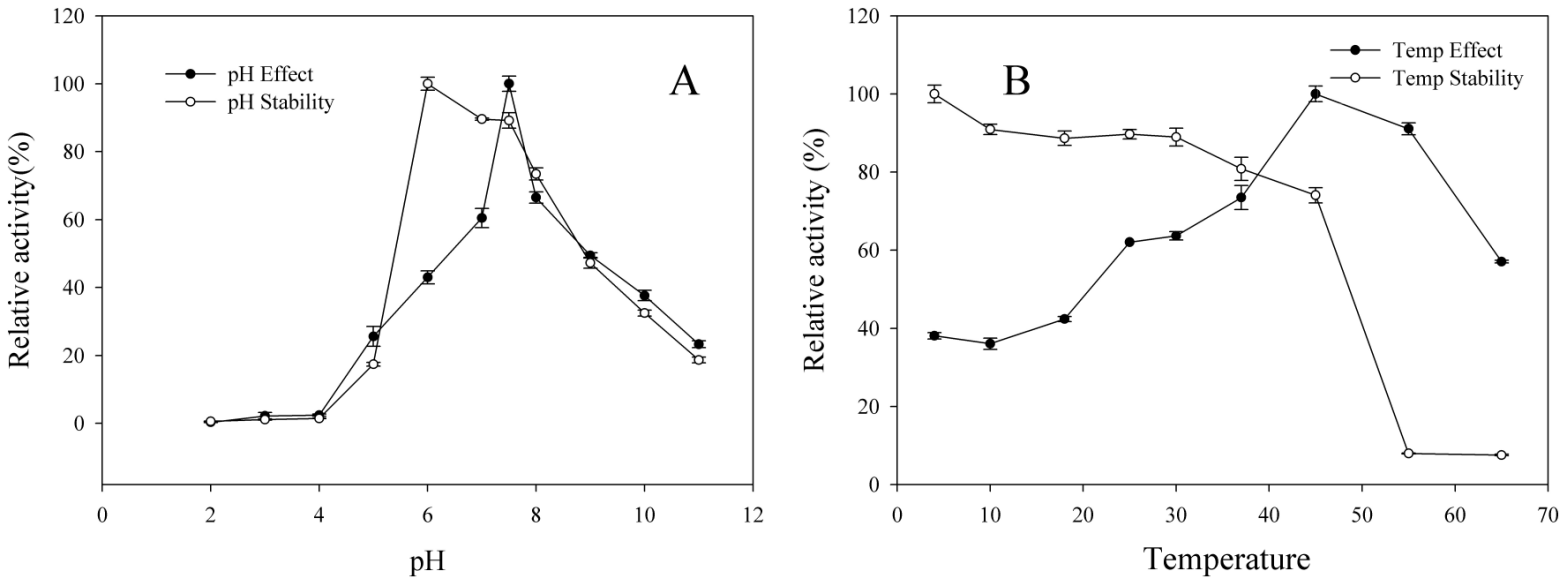
Effects of pH (A) and temperature (B) on the activity and stability of recombinant BglPm.

BglPm had optimal pH activity at 7.5 and stability at pH 6.0 to 7.5 in a sodium phosphate buffer at 37°C; from pH 8.0, the enzyme activity decreased swiftly, while at pH 5.0 the enzyme activity decreased to 17.4% (Fig. 3A). The optimal temperature activity was 45°C; at 30°C and 37°C, the enzyme has 63.7% and 73.5% of relative activity, respectively, while thermostability was decreased from 37°C and at 45°C the enzyme has 53.1% of relative activity, and not detected at 55°C (Fig. 3B). Though BglPm has optimum temperature at 45°C for pNPGlc, ginsenoside-conversion reaction was occurred at 37°C for extension of stable transformation activity.

The effects of metal ions, EDTA, β-mercaptoethanol, and SDS on BglQM activity were also investigated (Table 1). BglPm activity was not affected by β-mercaptoethanol, which is well known thiol group inhibitors. These results suggested that sulfhydryl groups may not be involved in the catalytic center of the enzyme. The enzyme was not affected by 1 mM and 10 mM Na^+^, K^+^, Mn^2+^, Ca^2+^, Zn^2+^, Co^2+^, and Cu^2+^. The chelating agent EDTA did not inhibit BglPm activity, which indicated that divalent cations are not required for enzymatic activity. The enzyme activity appeared to be 100% inhibited in the presence of sodium dodecyl-sulfate (SDS) and strongly inhibited in the presence of 10 mM Hg^2+^ and also inhibited in the presence of 10 mM Mg^2+^, which caused a 43% activity drop. However, no dramatic positive effects on the activity of the BglPm were found for the tested ion (Table 1).

**Table 1:** Effects of metal ions and chemical agents on the activity of purified recombinant BglPm.

	Relative activity	
	1 mM	10 mM
NaCl	98.8±2.5	100.3±1.4
KCl	99.0±1.3	100.2±2.2
MgCl_2_	88.3±0.9	57.0±4.7
MnCl_2_	87.1±2.2	126.0±6.6
CaCl_2_	94.6±3.6	85.9±1.2
ZnCl_2_	98.3±1.3	78.3±1.5
CoCl_2_	82.9±3.3	85.0±6.1
CuCl_2_	70.1±6.4	105.3±3.6
HgCl_2_	22.7±1.5	ND^a^
SDS	ND	ND
EDTA	102.6±5.4	86.3±4.8
β-Mercaptoethanol	101.5±6.3	86.5±7.1
DTT	96.8±3.5	80.14±4.2
Control	100±2.4	100±1.9

a Not determined.

The substrate specificity of BglPm was tested using 2.0 mM of p-nitrophenyl (pNP) and o-nitrophenyl (ONP)-glycosides with α and β configurations. The results showed that BglPm was only active against glucose moiety of pNP-β-D-glucopyranoside and oNP-β-D-glucopyranoside. It showed maximum activity towards pNP-β-D-glucopyranoside and 15% relative activity towards oNP-β-D-glucopyranoside.

### 3.4. Determination of kinetic parameters

The kinetic parameters of *V_max_* and *K_m_* of BglPm were determined by plotting the substrate concentration vs the initial velocity of each reaction and subjecting the data to no linear regression analysis. The *K_m_*, *V_max_*, *k_cat_* and *k_cat_*/*K_m_* values for 4 substrates were presented in Table 2. The catalytic efficiencies (*k_cat_*/*K_m_*) for pNPGlc, Rb_1_, Gyp XVII and Rd decreased in this order: pNPGlc (2.56±0.46 mM^−1^ S^−1^) > Rd (1.78±0.46 mM^−1^ S^−1^) > Rb_1_ (1.23±0.25 mM^−1^ S^−1^) > Gyp XVII (1.17±0.08 mM^−1^ S^−1^). BglPm has higher catalytic efficiency for Rd rather than Rb_1_ and Gyp XVII, which is attributed that the affinity for the outer glucose moiety at C20 of Rb_1_ may decrease. The catalytic efficiencies for ginsenosides is lower than that of β-glycosidase from *Sulfolobus acidocaldarius* [24] for ginsenosides Rd (4.8 mM^−1^ min^−1^) and Rb_1_ (4.8 mM^−1^ min^−1^).

**Table 2:** Kinetic parameters of recombinant BglPm on pNPGlc, Rb1, gypenoside XVII and Rd.

Substrate	*K_m_* (mM)	*V_max_* (µmol min^−1^ mg^−1^)	*k_cat_* (s^−1^)	*k_cat_*/*K_m_* (mM^−1^s^−1^)
pNPGlc	3.24±0.39	10.22±0.62	8.13±0.50	2.56±0.46
Rb_1_	0.384±0.064	0.369±0.014	0.458±0.018	1.23±0.25
Gypenoside XVII	0.415±0.024	0.391±0.006	0.487±0.007	1.17±0.08
Rd	0.352±0.053	0.484±0.053	0.601±0.066	1.78±0.46

### 3.5. Ginsenoside-transformation characteristics of BglPm

**Figure 4:**
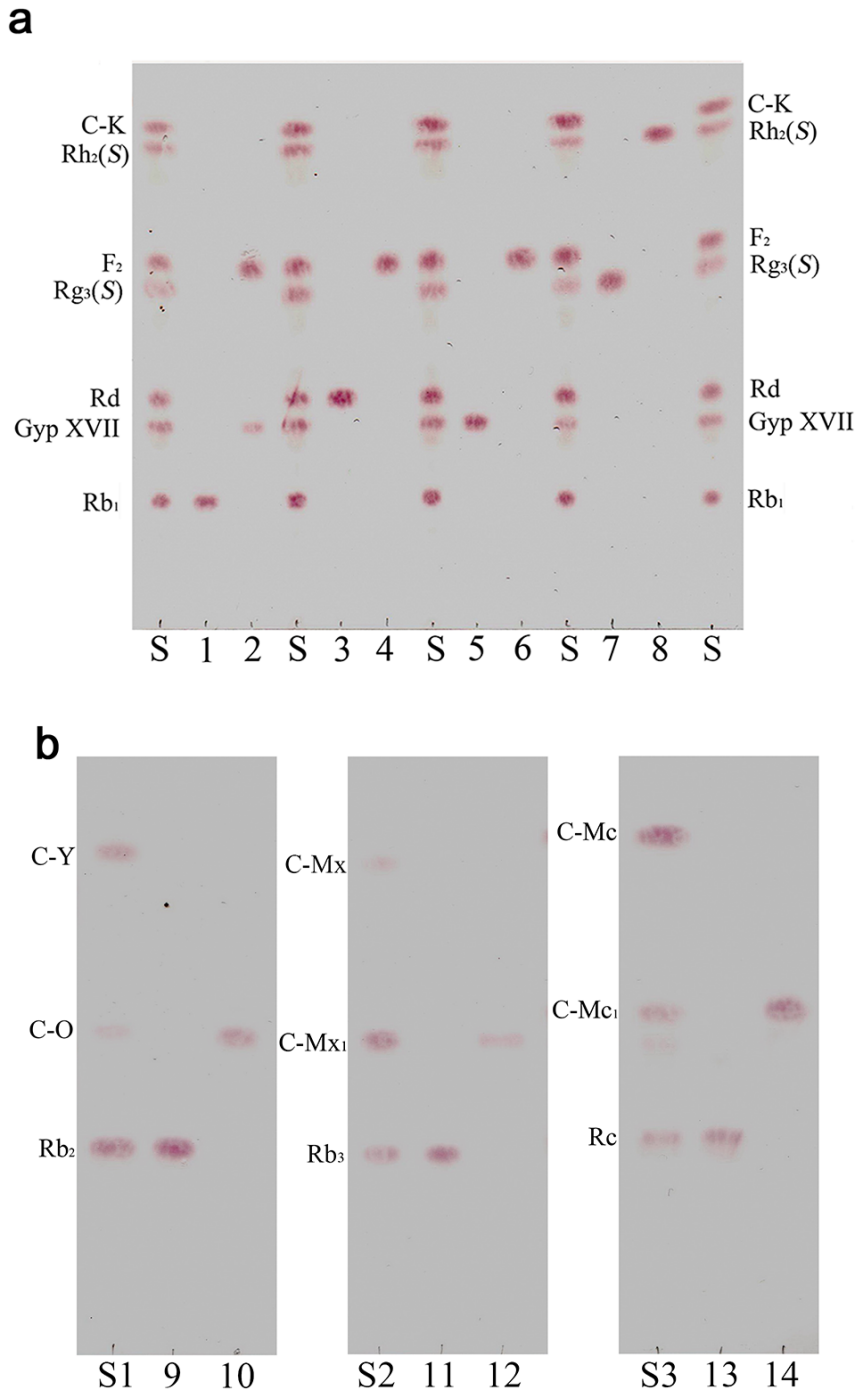
Thin layer chromatography (TLC) analyses of biotransformation of Rb_1_, Rd, Gyp XVII, Rg_3_(*S*) in (A) and Rb_2_, Rb_3_, Rc in (B) by recombinant BglPm. The reaction time was 30: CHCl_3_-CH_3_OH-H_2_O (65∶35∶10, lower phase). (S, S1, S2, S3), ginsenoside standards (PPD type ginsenoside mixtures); Standards: 1, 3, 5, 7, 9, 11, 13; Reaction mixture: 2, 4, 6, 8, 10, 12, 14; 1, Rb_1_; 2, reaction mixture of Rb_1_; 3, Rd; 4, reaction mixture of Rd; 5, Gyp XVII; 6, reaction mixture of Gyp XVII; 7, Rg_3_(*S*); 8, reaction mixture of Rg_3_(*S*); 9, Rb_2_; 10, reaction mixture of Rb_2_; 11, Rb_3_; 12, reaction mixture of Rb_3_; 13, Rc; 14, reaction mixture of Rc. Abbreviations: C-K, compound K.

**Figure 5:**
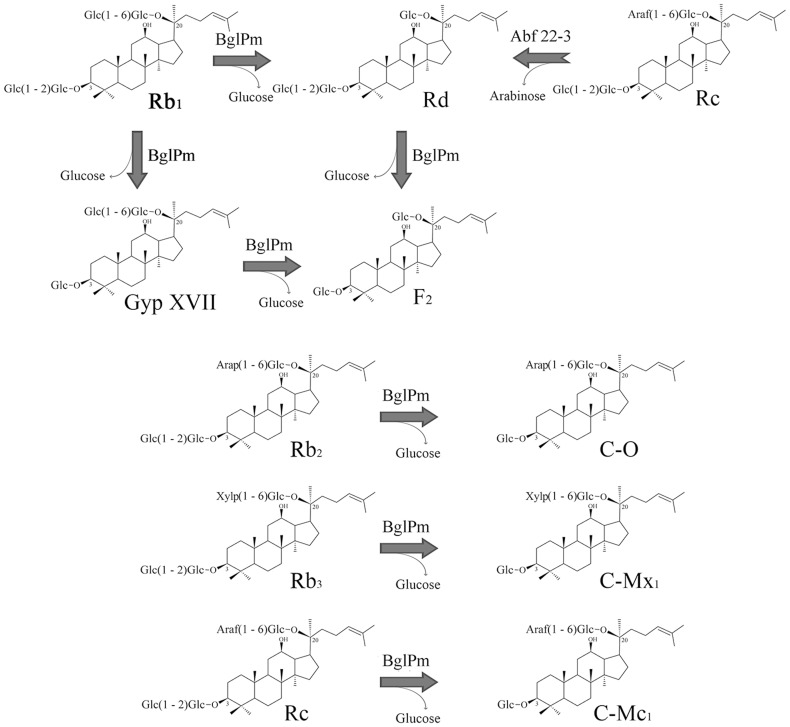
Biotransformation pathways of ginsenosides Rb_1_, Rd, Rb_2_, Rb_3_, and Rc by recombinant BglPm and incorporated pathways for F_2_ production by two glycoside hydrolases.

For the verification of the bioconversion pathways of the seven PPD type ginsenosides (Rb_1_, Rb_2_, Rb_3_, Rc, Rd, Gyp XVII and Rg_3_) by BglPm, the TLC analyses were performed at regular intervals. As shown Fig. 4, it is clear that the BglPm could transform seven ginsenosides (Rb_1_, Rb_2_, Rb_3_, Rc, Rd, Gyp XVII and Rg_3_) based on the R*_f_* values. The transformed ginsenosides were also determined using their retention times in the HPLC (data not shown). The proposed biotransformation pathways by BglPm for the PPD ginsenosides are as follows: Rb_1_ → Gyp XVII → F_2_; Rd → F_2_; Rg_3_(*S*) → Rh_2_(*S*); Rb_2_ → C-O; Rb_3_ → C-Mx_1_; Rc → C-Mc_1_ via the stepwise hydrolysis of the outer glucose moieties at the C3 position of aglycon (Fig. 5). BglPm hydrolyzes outer glucoses of C3 and C20 position of ginsenosides, similar to the β-glucosidase from *Sphingomonas* sp. 2F2 [25], but BglPm cannot hydrolyze inner glucose moiety of ginsenosides at C3 and C20.

A few ginsenoside hydrolyzing recombinant enzymes have been characterized to convert major ginsenosides to F_2_ (Table 3). A β-glucosidase from *Sulfolobus solfataricus* can hydrolyze Rb_1_ and Rd into F_2_, but this enzyme can continuously hydrolyze F_2_ into C-K [24]. A BglSp from *Sphingomonas* sp. 2F2 showed F_2_ production abilities from ginsenosides Rb_1_, Rb_2_, Rd, and Rc but the low conversion activity limited its application for F_2_ production [25] from PPDGM. Until now, the low transformation efficiencies and unconformable conversion pathways of recombinant glycosidase hydrolases limited the applications for the production F_2_ as gram-scale from PPDGM.

**Table 3:** Major ginsenoside transformation into F_2_ by the cloned glycoside hydrolases.

Glycoside hydrolase name	Microorganism	Ginsenoside conversion pathway	Glycoside hydrolase family	Reference
BglPm	*Paenibacillus mucilaginosus* KCTC3870^T^	Rb_1_→Gyp XVII→F_2_, Rd→F_2_	1	This study
β-glycosidase	*Sulfolobus solfataricus*	Rb_1_→Rd→F_2_→C-K, Rb_2_→Rd→F_2_→C-K, Rc→C-Mc→C-K	1	Noh et al, 2009
BglSp	*Sphingomonas* sp. 2F2	Rb_1_→ Gyp XVII→F_2_, Rb_2_→C-O→F_2_, Rc→C-Mc→F_2_, Rd→F_2_	1	Wang et al, 2011
BglF3	*Flavobacterium johnsoniae*	Rb_1_→Rd, Gyp XVII→F_2_	3	Hong et al, 2012
AbfA	*Rhodanobacter ginsenosidimutans* Gsoil 3054	C-Mc_1_→F_2_	51	An et al, 2011

### 3.6. Optimization of PPDGM concentration

**Figure 6:**
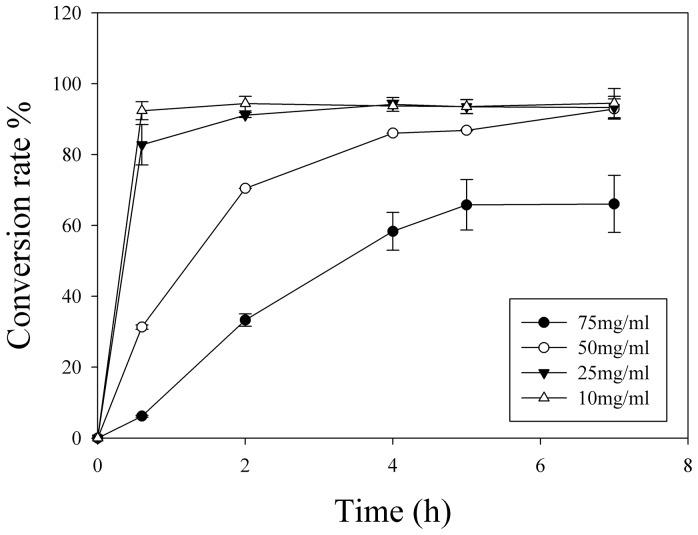
Effect of the concentration of PPDGM and crude BglPm on the production of ginsenoside F_2_.

F_2_ production efficiency of crude recombinant BglPm were tested with four PPDGM concentration(10 mg/ml, 25 mg/ml, 50 mg/ml, 75 mg/ml) in order to determine the appropriate substrate concentration for the decreasing reactor volume and economical enzyme concentration for reducing production costs. The time course of the ginsenosides (Rb_1,_ Rd, Gyp XVII, and F_2_) on the process was determined via HPLC analyses and the abundance of ginsenoside F_2_ was calculated (Fig. 6). In the test condition of a high substrate concentration (50 mg/ml), the ginsenosides mixture of Rb_1_ and Rd were completely converted ginsenoside F_2_ within 7 hours. The higher reaction condition (75 mg/ml) did not complete the conversion within 40 hours. Endowing the advantage to the smaller reactor size, the condition of a high concentration substrate (50 mg/ml) was adopted for the next scaled-up biotransformation step. Thus, these three reaction conditions were excluded in the next step.

### 3.7. Scaled-up production of ginsenosides F_2_

**Figure 7:**
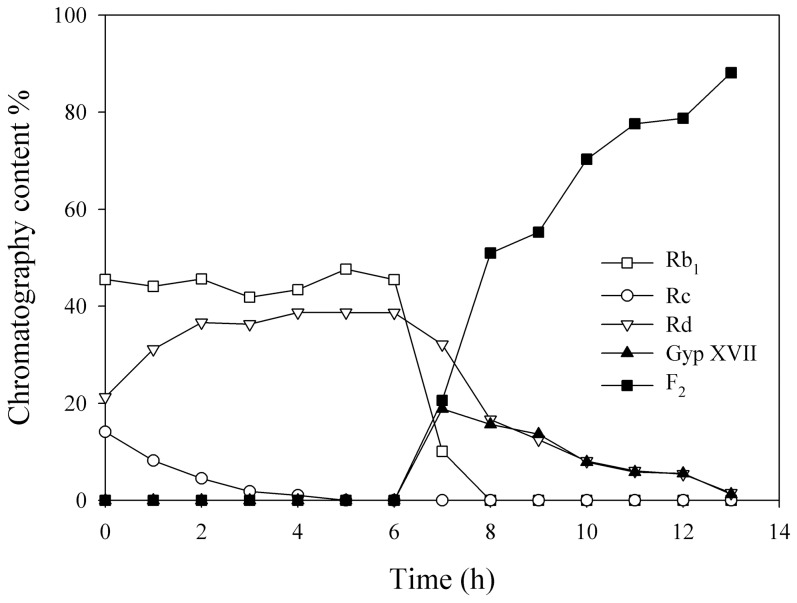
Relative abundance of ginsenosides Rb_1_, Rc, Rd, Gyp XVII and F_2_ in scaled-up production reactor in time course.

**Figure 8:**
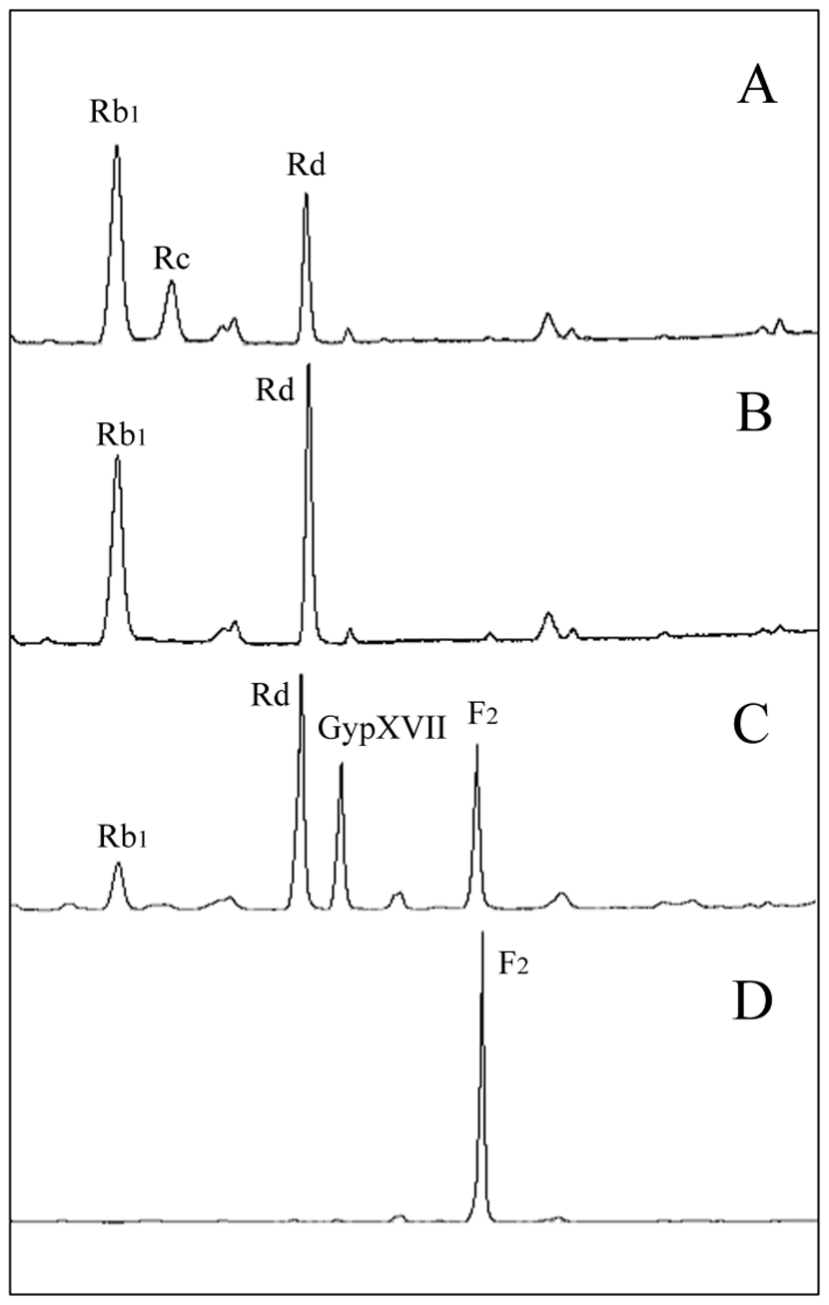
HPLC results of the transformation of PPDGM via Abf22-3 and followed by GST-BglPm; (a) ginsenoside peaks of PPDGM, (b) reaction mixture after 6 hour treatment with Abf-22-3, (c) reaction mixture after 1 hour treatment with GST-BglPm, (d) reaction mixture after 7 hour treatment with GST-BglPm. Abbreviations: PPDGM, protopanaxadiol type ginsenoside mixture.

PPDGM was used as substrate for the mass production of ginsenoside F_2_ with high purity because it is relatively abundant and can be efficiently separated in specialized ways from crude American ginseng extracts [26], [27]. The enzyme reaction occurred using the crude recombinant Abf22-3 followed by BglPm with PPDGM as the substrate with a concentration of 50 mg/ml as the final concentration in 5 L in order to produce F_2_ (Fig. 5). As shown in Fig. 7, the ginsenoside Rc was completely converted to Rd within six hours after the crude Abf22-3 was applied to the PPDGM. Thus, at this point, the crude lyophilized BglPm was applied to convert both the intact ginsenoside Rb_1_ and Rd in the PPDGM and transformed Rd via Abf22-3 to F_2_. The ginsenoside F_2_ was produced consecutively up to 7 hours after initiation until ginsenosides Rb_1_ and Rd were exhausted (Fig. 7). The reaction sample of each point were withdrawn and analyzed via HPLC, of which the chromatography images are shown in Fig. 8. It was demonstrated that when the bioconversion rate was nearly complete for ginsenoside Rb_1_, Rc and Rd which were not detected by the HPLC analysis (Fig. 8-D).

Among the PPDGM, ginsenosides Rb_2_ and Rb_3_ occupying approximately 7.6% (mole ratio) were not transformed by Abf22-3, but can be converted by BglPm, and metabolites C-O and C-Mx_1_ were remained in the solution (Fig. 8-D). The research team behind this paper has searched the ginsenoside hydrolyzing bacteria [28], [29] and constructed several ginsenoside-hydrolyzing recombinant enzymes [15], [21], [25], [30], [31], [32] through the process of surveying the ginsenoside-hydrolyzing enzymes. The finding of efficient BglPm that could efficiently convert Rb_1_ and Rd to F_2_ is a key factor in creating F_2_.

### 3.8. Purification of biotransformed F_2_

In order to remove the enzyme, salt, and free sugar from the reaction mixture of the 5 L reaction of PPDGM with Abf22-3 and BglPm, the mixture was centrifuged at 5,000 rpm for 15 min. Most of the ginsenoside F_2_ was precipitated to form a solid, with a small quantity remaining dissolved in the supernatant (data not shown). After purification step using column chromatography packed with HP20 resin, approximately 24 L of the 95% ethanol eluent was evaporated *in vacuo* in order to create 152 g of ginsenoside F_2_. Its chromatographic purity was 80.1% as determined via HPLC (Fig. 8). The impurities were ginsenosides C-O and C-Mx_1_ derived from the ginsenoside Rb_2_ and Rb_3_. As a result, 250 g of PPDGM was used as a substrate for biotransformation and 152 g of ginsenoside F_2_ with a purity of 80.1% was obtained. The PPDGM was primarily comprised of ginsenosides Rb_1_, Rc, and Rd in which ginsenosides Re, Rb_2_, Rb_3_, Rg_3_(*S*), and Rg_3_(*R*) were also included. Among these ginsenosides, the total molar amount of Rb_1_, Rc and Rd that could be biotransformed into F_2_ using the two recombinant enzymes (Abf22-3 and BglPm) was 162.2 mmol, which corresponds to 173.8 g of 250 g. The 76.2 g of residue was comprised of other types of ginsenosides and unknown impurities. The molar amount of the produced ginsenoside F_2_ was 155.2 mmol. This indicates that the recovery ratio through the biotransformation process using ginsenosides Rb_1_, Rc and Rd to F_2_ reached 95.7% during the entire bioprocess engineering. This is the first report of a cloned enzyme that is capable 100 gram-scale production of F_2_ through biotransformation.

## Conclusion

In this study, we identified and characterized a ginsenoside-transforming glycoside hydrolase (BglPm) from *Paenibacillus mucilaginosus*. The ginsenoside-transforming pathways and activities of BglPm were proper for mass production of minor ginsenoside F_2_ using relatively abundant PPDGM consisting of ginsenosides Rb_1_, Rc, and Rd. Combined usage with Rc-hydrolyzing α-L-arabinofuranosidase, 152 g of ginsenoside F_2_ with 80.1% chromatography purity was achieved from 250 g of PPDGM using BglPm. This enhanced production method offers an efficient method of preparing the minor ginsenoside F_2_ in a large scale to meet industrial needs.
